# The establishment and evaluation of a new model for the prediction of prostate cancer

**DOI:** 10.1097/MD.0000000000006138

**Published:** 2017-03-24

**Authors:** Qi Wang, Yan-Feng Li, Jun Jiang, Yong Zhang, Xu-Dong Liu, Ke Li

**Affiliations:** Department of Urology, Institute of Surgery Research, Daping Hospital, Third Military Medical University, Chongqing, China.

**Keywords:** prostate cancer, prostate cancer predictor, prostate-specific antigen

## Abstract

To develop a new prostate cancer predictor (PCP) model using the combination of total prostate-specific antigen (tPSA), free PSA (fPSA), and complexed PSA (cPSA).

The diagnoses of all the included patients were confirmed pathologically in Daping Hospital between December 1, 2011 and December 1, 2014. There were 54 PCa cases and 579 benign prostatic hyperplasia (BPH) cases with tPSA levels of 2 to 10 ng/mL, and 48 PCa cases and 147 BPH cases with tPSA levels of 10 to 20 ng/mL. Logistic regression and receiver operating characteristic curve (ROC) analyses were employed to compare the value of PCP (PCP = tPSA / fPSA × √cPSA) with tPSA, fPSA, the ratio of fPSA to tPSA (%fPSA), and cPSA for the differential diagnosis of PCa and BPH. Meanwhile, bootstrapping analysis was used to calculate the distribution and confidence intervals (CIs) for the area under the curve (AUC), and Hosmer–Lemeshow tests were used to calculate *P* values.

When tPSA levels were 2 to 10 ng/mL, the AUC of PCP (0.680) was significantly higher than that of tPSA (0.588), fPSA (0.571), %fPSA (0.675), and cPSA (0.613). When the sensitivity for the diagnosis of PCa was 90.7%, the specificity of PCP (22.8%) was higher than that of tPSA (11.1%), fPSA (11.2%), %fPSA (17.4%), and cPSA (15.5%). When tPSA levels were 10 to 20 ng/mL, the AUC of PCP (0.686) was significantly higher than that of tPSA (0.603), fPSA (0.643), %fPSA (0.679), and cPSA (0.647). When the sensitivity for the diagnosis of PCa was 91.7%, the specificity of PCP (29.3%) was higher than that of tPSA (10.9%), fPSA (10.2%), %fPSA (23.1%), and cPSA (18.4%).

PCP is a novel model for the prediction of PCa; it has more predictive value than tPSA, fPSA, %fPSA, and cPSA when tPSA levels are 2 to 20 ng/mL.

## 1 Introduction

PSA has played an important role in the early diagnosis, treatment strategy, and prognosis of prostate cancer (PCa).^[1]^ The widespread use of PSA has increased the detection rate of PCa significantly. Some countries or regions routinely perform PCa screening using PSA testing and have achieved certain results.^[2,3]^ However, PSA is a marker of prostate tissue and is not specific to PCa; therefore, the widespread application of PSA testing has inevitably resulted in a serious problem of over-diagnosis and over-treatment.^[4]^ To remedy this, %fPSA was applied clinically and significantly enhanced the detection rate of PCa in patients with tPSA levels of 4 to 10 ng/mL.^[5,6]^ Some studies also found that cPSA had a higher specificity than tPSA or %fPSA when the sensitivity for diagnosis of PCa was 95%,^[7]^ and it could be substituted for tPSA as the screening index for PCa.^[8]^

However, the detection rate of PCa remains at a very limited level using cPSA or %f PSA, and so many patients with BPH unnecessary undergo prostate biopsies. The current study combined tPSA, fPSA, and cPSA to develop and validate a new predictive model (tPSA/fPSA × √cPSA) for the diagnosis of PCa (PCP). The differential diagnostic value of PCP for PCa was evaluated by comparing the clinical application of PCP, tPSA, cPSA, and %fPSA. The aim was to improve the diagnostic accuracy of PCa and decrease the incidence of unnecessary prostate biopsies by the application of PCP.

## 2 Materials and methods

### 2.1 Study population

The diagnosis of all the included Chinese patients was confirmed pathologically in Daping Hospital between December 1, 2011 and December 1, 2014. There were 54 cases of PCa and 579 cases of BPH with tPSA levels of 2 to 10 ng/mL, and 48 cases of PCa and 147 cases of BPH with tPSA levels of 10 to 20 ng/mL. The decision to perform prostate biopsy was based on an increased tPSA, a low %fPSA, a suspicious digital rectum examination (DRE), or a suspicious transrectal ultrasonography (TRUS) according to the guidelines of Chinese Urological Association. Before serum PSA levels were tested, all factors that may cause an abnormal increase in serum PSA were investigated according to medical history, including bacterial acute prostatitis, hemophilia, cystoscopy, and the use of drugs that affect PSA levels.^[9]^ The research protocols and informed consents from patients were approved by the Ethics Committee of the Daping Hospital that belong to Third Military Medical University of Chongqing. All the research procedure was strictly executed in accordance with the Declaration of Helsinki.

### 2.2 Determining and calculating serum PSA levels

Before prostate biopsy or prostate surgery, blood samples were collected according to standard procedures.^[10]^ Serum tPSA and fPSA levels were determined using chemiluminescence with a Beckman Coulter Unicel DXI 800 automatic immunity analyzer. tPSA includes fPSA and cPSA, cPSA mainly consists of PSA-ACT (serum fPSA complexed with α-antitrypsin), and other forms of PSA in serum are too low to detect^[11,12]^; therefore, cPSA plus fPSA is generally equivalent to tPSA.

### 2.3 Analysis of pathological specimens

Attending urologists performed ultrasound-guided prostate biopsies according to a standardized scheme. Briefly, at least 12 biopsy cores were evenly taken from the top, intermediate region, and base of the prostate gland, and additional cores were taken when obviously abnormal areas were detected. Prostate biopsy specimens were placed in specific single-core specimen containers. Specimens were processed and evaluated by an experienced genitourinary pathologist. Prostate surgeries, including (transurethral resection of prostate, TURP) or laparoscopic radical prostatectomy, were performed according to the diagnosis and general condition of the patient. The pathological diagnosis of all was achieved and confirmed by the pathologists in our hospital.

### 2.4 Construction of the PCP model

The critical objective of the PCP is to achieve the best diagnosis of PCa, or achieve an area under the ROC curve (area under the curve [AUC]) for PCP that is the largest compared with tPSA, fPSA, %fPSA, and cPSA. A nonlinear prediction model was constructed using the combination of tPSA, fPSA, and cPSA: 
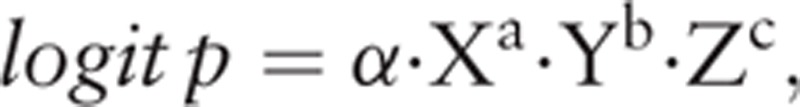


where α is the coefficient, X, Y, and Z represent tPSA, fPSA, and cPSA respectively, and a, b, and c are the power parameters of X, Y, and Z, respectively. Maximum likelihood estimates and iterative estimations were used to predict the unknown power parameter values of α, a, b, and c.^[13,14]^ Parameter sensitivity analysis was used to ensure the stability of the power parameter values.^[15]^ The power parameter values were added to the predicting model to obtain the PCP (PCP = tPSA/fPSA × √cPSA). Finally, logistic regressions were used to calculate the AUC of PCP for the diagnosis of PCa.

### 2.5 Statistical analysis

Student *t* tests were used to assess the normality of variables. Mann–Whitney *U* tests were used to compare not normally distributed continuous variables. Logistic regression was used to analyze the linear relationship between predictive variables and PCa. Hosmer–Lemeshow tests were used to assess the fitting goodness of the logistic models. Odds ratios (ORs) with 95% confidence intervals (CIs) were also calculated. ROC curves were used to quantify the predictive accuracy of the logistic models. To comprehensively demonstrate the predictive consequences of logistic models, bootstrapping analysis was used to calculate the distribution and CIs for AUCs, and Hosmer–Lemeshow tests were used to calculate *P* values.^[16]^

All data analyses were performed using SPSS version 13.0 (SPSS Inc, Chicago, IL), MedCalc Statistical Software version 15.2.2 (MedCalc Software, Ostend, Belgium), and R version 3.2.0 (The R Foundation for Statistical Computing). Higher *P* values in the Hosmer–Lemeshow test indicated that the tested models were more meaningful. The results of other analyses were considered significant with two-sided *P* value ≤0.05.

## 3 Results

### 3.1 The clinical characteristics of the study population

A total of 828 patients with tPSA levels of 2 to 20 ng/mL were included in this study. According to pathological reports, 102 (12.3%) patients had a final diagnosis of PCa. There was no obvious difference in the mean age of patients with PCa and BPH (71.7 vs 71.5 yr, respectively; *P* > 0.744). There was no significant difference in the median fPSA level between the 2 groups (1.0 vs 1.0 ng/mL, respectively; *P* > 0.572). Conversely, tPSA, cPSA, and PCP were significantly higher in the PCa group compared with the BPH group (9.2 vs 5.5 ng/mL, 8.0 vs 4.4 ng/mL, 22.1 vs 11.0, respectively; all *P* < 0.000). Conversely, %fPSA levels were significantly lower in the PCa group than in the BPH group (0.13 vs 0.19, *P* < 0.000) (Table 1).

**Table 1:**
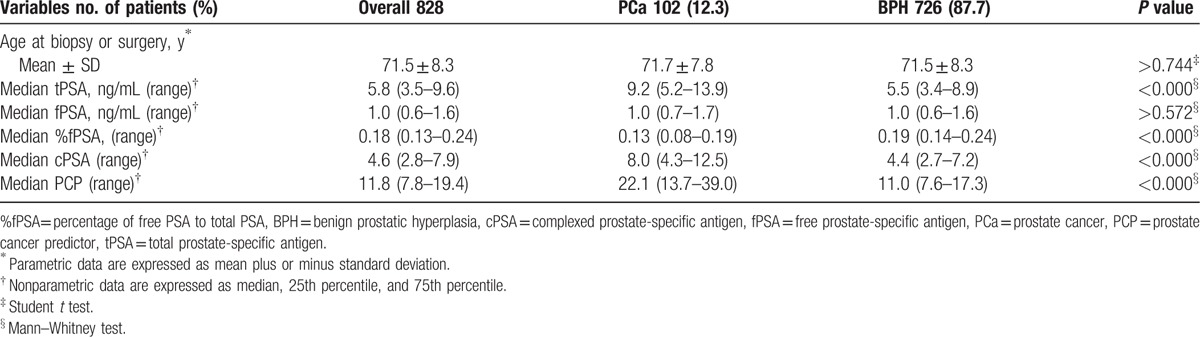
Descriptive characteristics of the study population.

### 3.2 The PSA indexes and PCP's abilities to predict PCa

**Figure 1:**
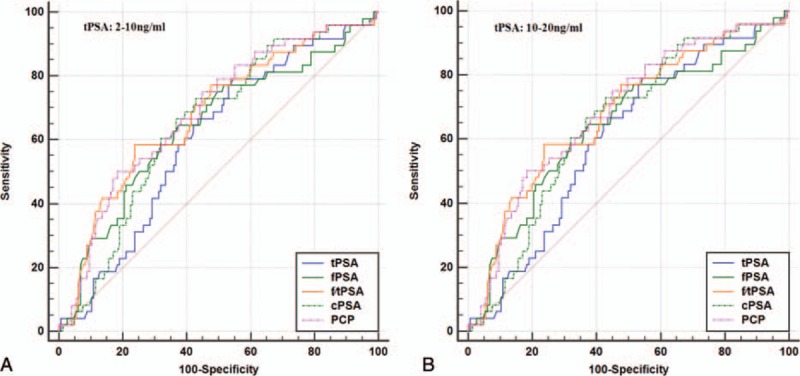
Receiver operating characteristic curves show the accuracy of individual predictors for predicting prostate cancer. %fPSA = percentage of free PSA to total PSA, cPSA = complexed prostate-specific antigen, fPSA = free prostate-specific antigen, PCP = prostate cancer predictor, PSA = prostate-specific antigen, tPSA = total prostate-specific antigen.

**Figure 2:**
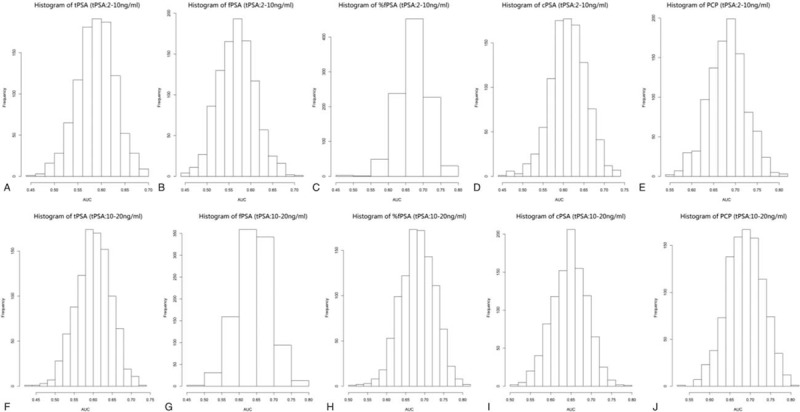
Histograms showing the distribution of individual predictors with their AUC. %fPSA = percentage of free PSA to total PSA, cPSA = complexed prostate-specific antigen, fPSA = free prostate-specific antigen, PCP = prostate cancer predictor, PSA = prostate-specific antigen, tPSA = total prostate-specific antigen.

**Figure 3:**
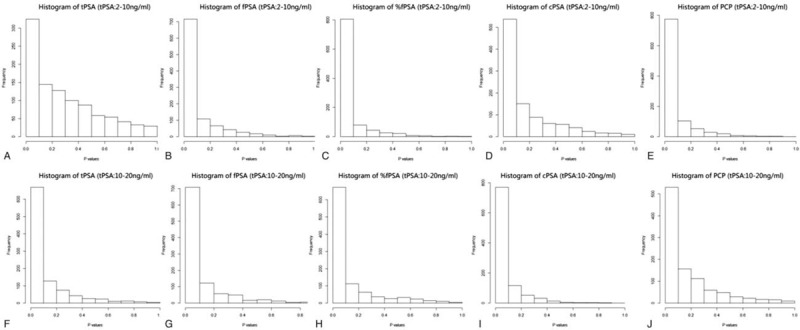
Histograms showing the distribution of Hosmer–Lemeshow tests with the resulting *P* values. %fPSA = percentage of free PSA to total PSA, cPSA = complexed prostate-specific antigen, fPSA = free prostate-specific antigen, PCP = prostate cancer predictor, PSA = prostate-specific antigen, tPSA = total prostate-specific antigen.

When tPSA levels were 2 to 10 ng/mL, univariate logistic regression analysis showed that PCP (*P* = 0.000), cPSA (*P* = 0.002), %fPSA (*P* = 0.000), and tPSA (*P* = 0.024) were all significantly related to PCa (Table 2), and that the correlation between PCP and %fPSA with PCa were closer than the correlation between tPSA or cPSA and PCa. In contrast, fPSA (*P* = 0.079) had no obvious correlation with PCa. In ROC curve analysis, the AUC of PCP was 0.680, which was significantly higher than that of tPSA (0.588), fPSA (0.571), %fPSA (0.675), and cPSA (0.613) (Fig. 1A). Next, bootstrapping was used to reanalyze the data and demonstrate the distribution and CIs for the AUC, and Hosmer–Lemeshow tests were used to calculate *P* values (Table 3, Figs. 2A–E, 3A–E). The results indicated that PCP was the most accurate predictor of PCa, followed by %fPSA and cPSA.

**Table 2:**
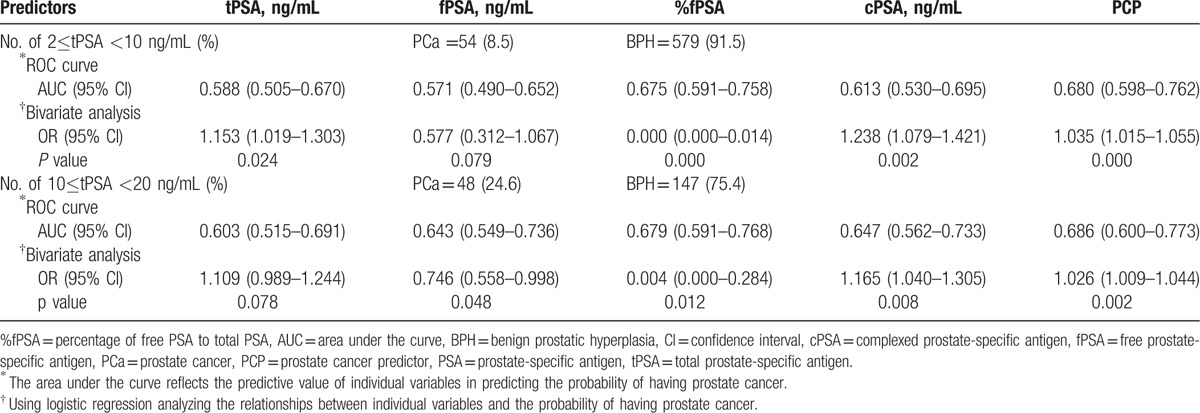
Logestic regression and ROC analysis predicting the probability of PCa was set at (I) 2≤tPSA <10 ng/mL, (II) 10≤tPSA <20 ng/mL.

**Table 3:**
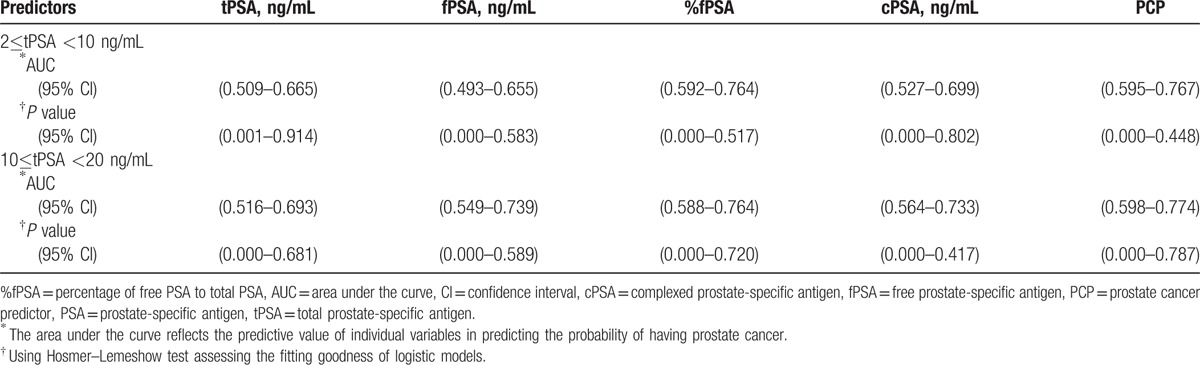
Bootstrapping analysis calculating the CIs for AUC and Hosmer–Lemeshow test with its *P* value was set at (I) 2≤tPSA <10 ng/mL, (II) 10≤tPSA <20 ng/mL.

When tPSA levels were 10 to 20 ng/mL, univariate logistic regression analysis showed that PCP (*P* = 0.002), cPSA (*P* = 0.008), %fPSA (*P* = 0.012), and fPSA (*P* = 0.048) were significantly associated with PCa (Table 2). In contrast, tPSA (*P* = 0.078) was not significantly associated with PCa. There was a closer correlation between PCP and PCa than %fPSA or cPSA and PCa. In contrast, fPSA (*P* = 0.048) was only weakly correlated with PCa, and tPSA (*P* = 0.078) had no obvious correlation with PCa. In ROC curve analysis, the AUC of PCP was 0.686, which is significantly higher than that of tPSA (0.603), fPSA (0.643), %f PSA (0.679), and cPSA (0.647) (Fig. 1B). Next, bootstrapping was performed to reanalyze the data and determine the distribution and CIs for the AUC, and Hosmer–Lemeshow tests were used to calculate *P* values (Table 3, Figs. 2F–J, 3F–J). This univariate accuracy analysis indicated that PCP was the most accurate predictor of PCa, followed by %fPSA and cPSA.

### 3.3 The PSA indexes and PCP's sensitivities and specificities for prediction of PCa

When tPSA levels were 2 to 10 ng/mL and the sensitivity for the diagnosis of PCa was 90.7%, the specificity of PCP was 22.8%, which is significantly higher than that of tPSA (11.1%), fPSA (11.2%), %fPSA (17.4%), and cPSA (15.5%). If the specificity for the diagnosis of PCa was ∼90%, the sensitivity of PCP (33.3%) remained significantly higher than that of tPSA (22.2%), fPSA (20.4%), %fPSA (29.6%), and cPSA (20.4%) (Table 4).

**Table 4:**
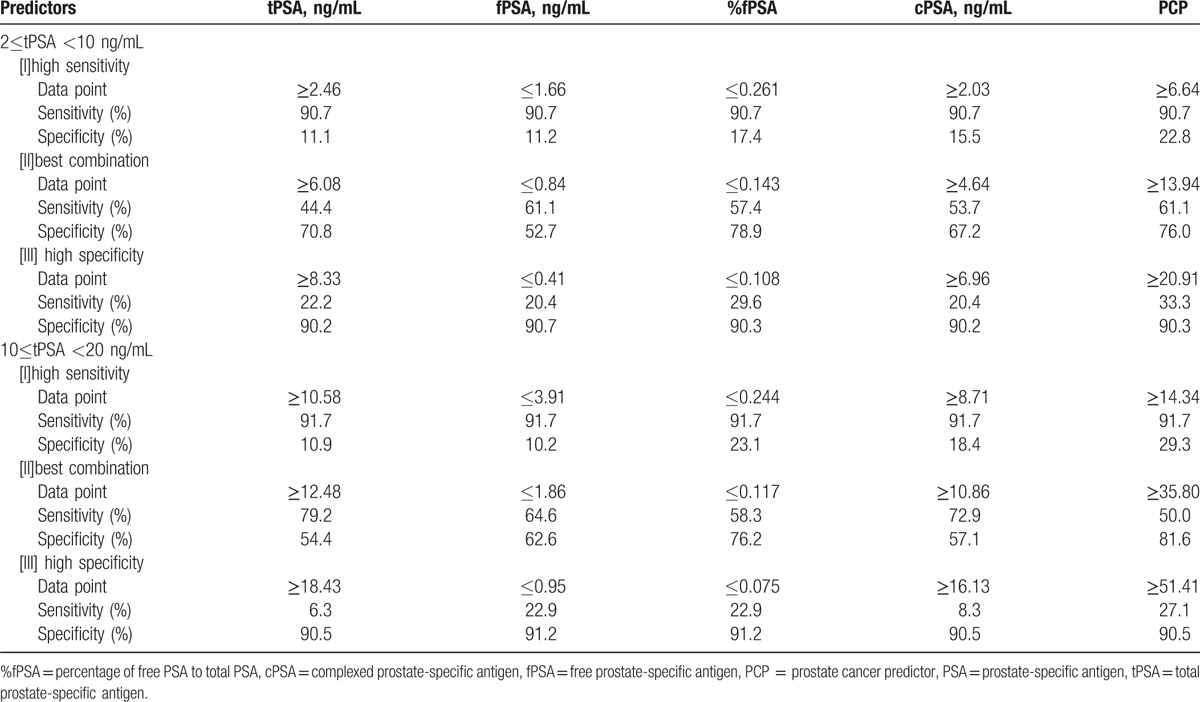
Three levels of predictive variables for prediction of prostate cancer: (I) high sensitivity, (II) best balance of sensitivity and specificity, (III) high specificity.

When tPSA levels were 10 to 20 ng/mL and the sensitivity for diagnosis of PCa was 91.7%, the specificity of PCP (29.3%) was significantly higher than that of tPSA (10.9%), fPSA (10.2%), %fPSA (23.1%), and cPSA (18.4%). If the specificity for the diagnosis of PCa was ∼91%, the sensitivity of PCP (27.1%) was significantly higher than that of tPSA (6.3%), fPSA (22.9%), %fPSA (22.9%), and cPSA (8.3%) (Table 4).

## 4 Discussions

PCa is one of the most common urogenital tumors in elderly men, particularly in Western countries.^[17]^ Although tPSA and %fPSA, which have the advantages of being simple, efficient, and rapid, are currently the most important serum markers for the diagnosis of PCa, many patients without PCa still undergo unnecessary prostate biopsy. Therefore, more accurate predictive markers are urgently needed.^[18]^ Novel predictive methods or tools for the early diagnosis of PCa are discovered regularly, but a variety of deficiencies have so far limited the promotion and application of these clinically. Larne et al^[19]^ built a PCa predicting tool by detecting the levels of 4 different microRNAs, and demonstrated that the tool was more efficient than PSA. However, no single microRNA has been applied to the clinical diagnosis of PCa at present; thus, the efficiency of the tool for the diagnosis of PCa remains questionable. Finne et al^[20]^ constructed a multivariate linear model and drew a nomogram using logistic regression analysis based on tPSA, %fPSA, prostate volume, and a digital rectal examination (DRE). The analysis showed that this model could decrease the false-positive rate more efficiently than %fPSA or prostate-specific antigen density (PSAD). However, prostate volume and DRE measurements were strongly associated with the physician's judgment and operating ability, which may affect the stability of the model for predicting PCa. Stephan et al^[21]^ created an artificial neural network (ANN) by analyzing tPSA, fPSA, macrophage inhibitory cytokine 1 (MIC-1), human kallikrein 11 (hK11), and migration inhibitor factor (MIF). Unfortunately, the specificity of the tool for diagnosing PCa was only better than %fPSA when the sensitivity was 90% or 95%, which is far from satisfactory. Auprich et al^[22]^ reported that prostate cancer antigen-3 had a greater ability to predict PCa than tPSA and %fPSA. Further, prostate cancer antigen-3 could be included in existing risk-stratifying tools or nomograms to help determine whether a patient should undergo prostate biopsy. However, measuring prostate cancer antigen-3 is more complex and incurs a higher cost, which limits its application in clinical practice. Stephan et al^[23]^ found that an artificial neural network based on precursor PSA and %fPSA had the same effect as an artificial neural network composed of tPSA, %fPSA, age, prostate volume, and DRE. However, the specificity of these tools for predicting PCa was not improved significantly compared with %fPSA when the sensitivity was 95%. Lughezzani et al^[24]^ developed a nomogram based on age, prostate volume, DRE, and prostate health index, and discovered that the nomogram was better than tPSA, %fPSA, and prostate health index for the prediction of PCa. Meanwhile, using only the prostate health index was better than tPSA or %fPSA. However, the prostate health index consisted of [-2]proPSA (a stable form of proenzyme PSA), tPSA, and fPSA; therefore, detecting [-2]proPSA requires an additional procedure to simple serum PSA tests, which undoubtedly increases the economic costs.

To search for a better predictive tool for the diagnosis of PCa, the current study successfully developed a new prostate cancer predictor (PCP) model based on the existing index of serum PSA through mathematical modeling, parameter calculations, and stability verifying. The current model has many advantages compared with previous studies. First, this is the first time that a nonlinear exponential function model has been constructed for the early diagnosis of PCa by combing tPSA, fPSA, and cPSA. Second, since the variables contained in the PCP are available from conventional serum PSA tests, the technology of detecting each variable was very mature and the PCP model did not need additional items to be tested. Third, nonobjective results caused by artificial judgment differences could be avoided because each variable was tested using automatic equipment.

tPSA and %fPSA still are the most widely used early clinical predictors of PCa and they provide the essential evidence to determine the need for prostate biopsy. The results of the present study suggest that PCP is significantly correlated with the incidence of PCa when tPSA levels are 2 to 10 ng/mL or 10 to 20 ng/mL, and could predict PCa more accurately than tPSA, fPSA, cPSA, or %fPSA. When tPSA levels were 2 to 10 ng/mL and the sensitivity for the diagnosis of PCa was 90.7% the specificity of PCP was 22.8%, which was 11.7%, 11.6%, 5.4%, and 7.3% higher than tPSA, fPSA, %fPSA, and cPSA, respectively. When tPSA levels were 10 to 20 ng/mL and the sensitivity for the diagnosis of PCa was 91.7%, the specificity of PCP for the diagnosis of PCa was 29.3%, which was 18.4%, 19.1%, 6.2%, and 10.9% higher than tPSA, fPSA, %fPSA, and cPSA, respectively. The results of this study confirm that PCP is the most accurate predictor of the presence of PCa compared with tPSA, fPSA, %fPSA, or cPSA in patients with tPSA levels of 2 to 20 ng/mL. The clinical application of PCP could avoid more unnecessary biopsies without missing significant cases of cancer.

In summary, PCP had more predictive value than tPSA, fPSA, %fPSA, and cPSA when tPSA levels were 2 to 20 ng/mL. This suggests that PCP could further reduce unnecessary prostate biopsies and the risk of over-diagnosis and over-treatment. Nevertheless, the value of PCP in combination with other indicators still requires further study. If the value of PCP for predicting PCa is accepted, this model is likely to replace tPSA or %fPSA as a novel clinical screening tool for PCa.

## Acknowledgment

The authors deeply appreciate Dr Zhi-Bing Deng, the Director of Statistical Analysis at the Scientific Research Data Center in the West China College of Medicine, Sichuan University, who assisted with the completion of statistics and drawing of histograms.
